# Melanin pigmentation variations in the larval cuticle of almond moth, *Ephestia cautella* caused by gamma radiation

**DOI:** 10.1038/s41598-023-48154-w

**Published:** 2023-11-29

**Authors:** Ali Hamza, Nagwan Zahran

**Affiliations:** Department of Natural Products Research, National Center for Radiation Research and Technology (NCRRT), Egyptian Atomic Energy Authority (EAEA), Cairo, Egypt

**Keywords:** Physiology, Zoology

## Abstract

To determine the effects of gamma radiation on the melanization process and phenoloxidase activity, *Ephestia cautella* larvae were exposed to dosages of 200, 400, 600, 800, and 1000 Gy. After irradiation, the number of non melanized larvae and the number exhibiting a slight melanization usually increased. The degree of melanization in treated larvae differed significantly from untreated larvae. The amount of melanin usually decreases as the dosage increases and as time passes after the treatment. The results of the phenoloxidase assay indicate that the enzyme activity responds differently to radiation. For instance, at doses of 200, 400, and 800 Gy, the enzyme activity remained consistent in both control and irradiated larvae. However, at doses of 600 and 1000 Gy, the enzyme activity increased to 14.92 and 13.37 O.D. units, respectively, compared to 8.81 O.D. units in the control. In order to determine if irradiated larvae have been previously exposed to ionizing radiation, a quick and easy test based on phenoloxidase activity or the melanization response is presented for use in quarantine treatment. Histological changes, specifically in the pigment granules of melanin, were studied using a light microscope. Upon inspection of the unirradiated larvae, it was observed that brown melanin pigment granules were deposited in the epicuticle and exocuticle layers of the cuticle. When gamma radiation dosages were administered to larvae, it was observed that the melanin pigment gradually diminished until it vanished at the highest dose (1000 Gy).

## Introduction

The almond moth, *Ephestia cautella* (Lepidoptera: pyralidae) is a particularly devastating pest of storage commodities in warmer climate and it also known as tropical ware-house moth, fig moth and flour moth^1^. The moth attacks variety of stored products including rice, wheat, maize, beans, cotton seeds, flours and brans. It also fed on dried fruits like figs, almond, dates, pears, groundnut, walnuts, cocoa and tobacco^2^.Only the larval stage of the insect is responsible for the majority of the infestation. The remainder of the kernel is unharmed as the larvae mostly consume the germ part. In cases of bulk infestation, its damage is limited to peripheral top layers only. Web formation covers the bags, floor space and mill machinery thereby causing clogging in the mills.

Irradiation of agricultural commodities is an effective quarantine measure and disinfestation method against stored product pests. The irradiation can be used for disinfesting a wide variety of agricultural products and materials^3^. Pests do not instantly die at the low doses of ionizing radiation recommended for quarantine treatment^4^. It is essential to confirm that any insects that survive the radiation treatment cannot reproduce or spread to new areas, particularly in products that have been radioactively sterilized for quarantine purposes.

Melanization is defined as a process by which the cuticle becomes dark and sometimes can be in normally non melanized cuticle by mechanical injury which somehow triggers tyrosinase activity^5^.

It is simple to measure phenoloxidase, which is present in all insects. The melanization of insect cuticle is greatly aided by the enzyme. By converting aromatic quinones and their byproducts into melanin, the requisite pigments for the cuticle would be produced^6^.

Insects frequently contain melanin, which gives them their red to brown and black colors. Melanin production is a multi-step, enzymatically regulated process that is catalyzed by several kinds of phenoloxidase.

Following wounding, it happens in many insects (wound healing)^7^, during encapsulation of foreign objects^8,9^, tanning, pigmentation and after death. According to Nation et al.^10^ irradiation-caused suppression of the darkening or melanization that often follows death or injury in a living insect may be a useful signal for identifying irradiated insects. Supawan et al.^11^ reported the degree of melanization in non-irradiated *Callosobruchus chinensis* larvae to be significantly different from the irradiated larvae.

One of the most intricate biological composites is the cuticle of insects. It is well recognised to have a spatial hierarchical architecture, which can have multiple layers at the lowest level of hierarchy. A simplified insect cuticle traditionally consists of three layers^12^: (i) epicuticle, (ii) exocuticle, and (iii) endocuticle. Epicuticle is the outermost layer that is usually thin and has a cement-like chitin-lacking structure^13^. The other two layers, in contrast, have lamellar organizations and contain chitin^14^. Exocuticle is a layer that is rigid and heavily sclerotized^15^. The exocuticle's sublayers are dense and typically take the form of a three-dimensional (3D) helicoidal arrangement^16^. However, the endocuticle is softer, less thick, more hydrated, and frequently resilin-bearing^17,18^. In addition to offering crucial structural support, the exoskeleton of insects serves as a canvas for the deposit of pigments or the sculpting of structural colors that are employed for mate signaling and camouflage.

The skeleton is made up of cuticle, an extracellular matrix made up of chitin fibers, cuticular proteins, lipids, and colors^19^. Melanin is one of the most prevalent pigments in insect cuticles, and many products of the melanin pathway also contribute to cuticle hardening/sclerotization, making the processes of cuticular melanization and cuticular sclerotization closely related in insects. For instance, cuticle rigidity is brought about by the cross-linking of molecules in the melanin pathway with nucleophilic amino acid residues and cuticular proteins^20–23^. The building of the insect exoskeleton and the coloration of the insect depend on the interaction between all these cuticle components^24^.

At a quarantine station, it can be challenging to determine if living larvae have undergone irradiation treatment and if it has received the required dose. This is why it's necessary to have methods in place to detect whether insect pests have been previously exposed to ionizing radiation. One of these ways is the post-radiation inhibition of the melanization process. Therefore, the purpose of this research is to use a method for the identification of irradiated insect larvae based on the post-radiation inhibition of the melanization process.

## Materials and methods

### Insect culture

*Ephestia cautella*, the almond moth, has been maintained in standard laboratory culture at the Department of Natural Products Research, National Center for Radiation Research and Technology (NCRRT), Egyptian Atomic Energy Authority (EAEA), Cairo, Egypt. The Almond moth larvae were kept in a mixture that contained (65% broken wheat, 10% sugar, 15% glycerin, and 10% brewer's yeast to 1 kg of media), at 26 °C and 70°R.H.

### Irradiation technique

The 4th instar larvae (20 days after egg hatch) were divided into groups of 15 and exposed to radiation doses of 200, 400, 600, 800, and 1000 Gy. The control group of 4th instar larvae wasn’t irradiated. The irradiation technique was carried out by a Gamma Cell (Co^60^ source) irradiation unit Model 220 located at National Center for Radiation Research and Technology (NCRRT). At the time of the experimental research, the dose rate was 1.107 KGy/hour.

### Measurement of melanization

Fourth instar larvae, both irradiated and unirradiated, were placed in Petri dishes with a rearing medium under controlled laboratory conditions. Larvae from each treatment group were selected after one week to assess cuticle melanization. The chosen larvae were first kept in a freezer (− 4 °C) for 2 h to kill them. After that, the larvae were taken out of the freezer and placed on a white background at room temperature for observation. The melanization of these larvae was evaluated visually and recorded photographically after 24 h.

An index of melanization was calculated using the method described by Ignatowicz and Lupa^25^ as a mean percentage of larval body encompassed by darkening. Irradiated larvae were compared to unirradiated larvae in terms of melanization inhibition. To determine the index value, the body was divided into three parts. One-tenth of the body was dark gray, two-tenths were of normal color, and six-tenths were black. For example, when melanization was inhibited in 66.6% of the body, the index value was calculated as 0.666 (1/10 × 0.66 + 2/10 × 0.0 + 6/10 × 1.00)^25^.

### Phenoloxidase activity

The 4^th^ instar larvae were exposed to doses ranging from 200 to 1000 Gy of gamma radiation. Afterward, they were placed in petri dishes containing a rearing medium and returned to the rearing room. Larvae were removed from the media after one week and to remove food particles from them, they were washed with tap water in a small dish, then placed on filter paper, and dried before examining the enzyme activity. Phenoloxidase activity was determined according to a modification of Ishaaya^26^, in a reaction mixture consisting of 0.5 ml phosphate buffer (0.1 M, pH7), 200 µl enzyme solution and 200 µl catechol solution (2%). The substrate and other components of the reaction mixture were individually incubated at the reaction's optimum temperature (25 °C) prior to the initiation of the reaction.

Catechol solution was added to start the enzyme reaction. Then after exactly 1 min, the optical density was determined. Zero adjustment was against sample blank at 405 nm. Bovine albumin standard was purchased from Stanbio laboratory (Texas, USA). Commasie brilliant blue G-250 was from sigma (sigma chemical co.). P- nitroanisole (purity 97%) was obtained from Ubichem Ltd. (Ham pshire), while nicotinamide ademine dinucleotide phosphate (reduced form, NADPH) was from BDH chemicals Ltd. (Poole, England). The remaining chemicals were of high grade and were bought from commercial local companies.

For biochemical analysis, larvae were homogenized in a chilled glass Teflon tissue homogenizer (ST–2 Mechanic-Preczyina, Poland). Supernatants were homogenized and then stored at − 20 °C in a deep freezer until they were used for biochemical tests. The absorbance of colored chemicals or metabolic products was measured using a double beam ultraviolet/visible spectrophotometer (spectronic 1201, Milton Roy Co., USA).

The larvae were prepared as described by Amin^27^. They were homogenized in distilled water (50 mg/1 ml). Homogenates were centrifuged at 8000 r.p.m. for 15 min at 2 °C in a refrigerated centrifuge. The deposits were thrown away, and the supernatants, also known as enzyme extract, can be kept for at least a week at 50 °C without significantly losing activity.

### Histological studies

Pieces of cuticle from the dorsal abdominal segment (between the first and second prolegs) of 4^th^ instar larvae of *E. cautella*, were dissected in Ringer physiological solution for histological studies, and after that fixation were conducted in FAA (formalin acetic alcohol) for 2 days. The process of dehydration involved ascending graded series of ethanol for 12 h, followed by absolute ethanol for 10 h. Samples were cleared first in an absolute ethanol and xylene (1:1) combination for two days, then in pure xylene overnight in a 65 °C oven, then specimens embedded in pure melted paraffin wax (mp 58–60 °C) over night. Samples were blocked in the suitable orientation. Using a manual microtone, serial longitudinal and cross sections were cut at a thickness of 8u. Ribbons were mounted and adhered with Mayers media (egg albumin and glycerin (1:1). The sections were stained with Ehrlich shaematoxyline, and counterskined in eosin technique. The stained sections were examined and photographed by a light microscope Sawires^28^.

### Statistics

All experiments contained 3–4 replicates (insects homogenates), and the results of biochemical determinations were pooled from triplicate determinations. The results were analyzed by one – way analysis of variance (ANOVA) using Costat statistical software (cohort software, Berkeley). When the ANOVA statistics were significant (P < 0.01), means were compared by the Duncan’s multiple range test.

## Results and disscussion

### Melanin measurement in unirradiated and irradiated 4th instar larvae of Almond moth, *Ephestia cautella*

**Figure 1:**
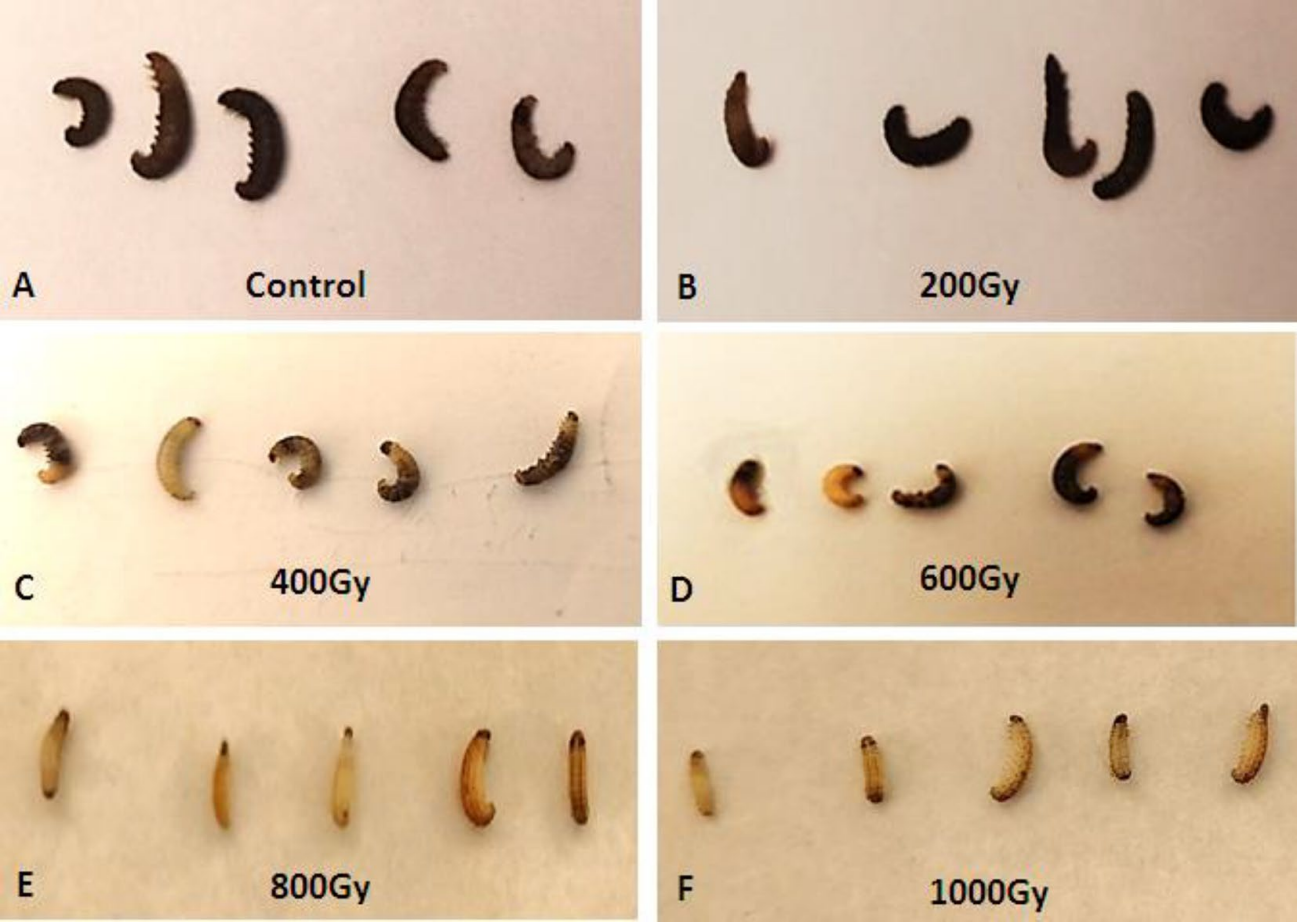
Degrees of darkening in the 4th instar larvae of *Ephestia cautella* after irradiated with different gamma doses.

Unirradiated 4th instar larvae of *Ephestia cautella* showed a strong melanization after killing by freezing. Figure 1 presents color of *E. cautella* larvae at different doses of gamma radiation. When the 4th instar larvae were irradiated with various doses (0, 200, 400, 600, 800 and 1000 Gy) and killed by freezing after time elapsed one week from the exposure to the gamma radiation, the control larvae were extensively melanized and then turned black (Fig. 1A). The degree of melanization in irradiated larvae was significantly different from unirradiated larvae, generally it decreased with increasing dose. Larvae irradiated with 200 Gy showed similar melanization to the control, although the black colour grew more slowly (Fig. 1B). Larvae irradiated with 400 and 600 Gy were intermediate between dark gray and slight gray (Fig. 1C,D). Most of the irradiated larvae with 800 Gy showed a clear lack of melanization (natural color) or melanized only partially and and they appeared to be smaller than control larvae (Fig. 1E). the highest suppression of melanization was recorded in larvae irradiated with a 1000 Gy, the larvae were just completely natural color (creamy) (Fig. 1F).

### Index of melanization for unirradiated and irradiated larvae of *Ephestia cautella*

The control 4th instar larvae of *E. cautella* exhibited a darkening of their body However, a high value of melanin index (100%) was found for the control (Table 1). The number of non melanized larvae and larvae exhibiting a slight melanization usually increased after the exposure of radiation doses. The darkening of larvae killed by freezing after one week of irradiation with 200, 400, 600, 800 and 1000 Gy decreased from 100% in control to 93.2, 57.2, 37.4, 26.4 and 0%, respectively.

**Table 1:** Index of melanization (percentage of body encompassed by melanization) for unirradiated and irradiated larvae of *Ephestia cautella*.

Dose (Gy)	Index of melanization (%) (Percentage of body encompassed by melanization)*
Control	100^a^
200	93.2^a^
400	57.2^b^
600	37.4^bc^
800	26.4^c^
1000	0^d^
F value	25.62
L.S.D. 5%	22.48

*Common letter following the mean indicates no significant difference between means in a column.

In general, all radiation doses had substantially lower levels of melanin than the control and the dose of 200 Gy, indicating a clear inhibition effect of irradiation on the process. (Table 1).

### Phenoloxidase activity in unirradiated and irradiated larvae of *Ephestia cautella*

**Figure 2:**
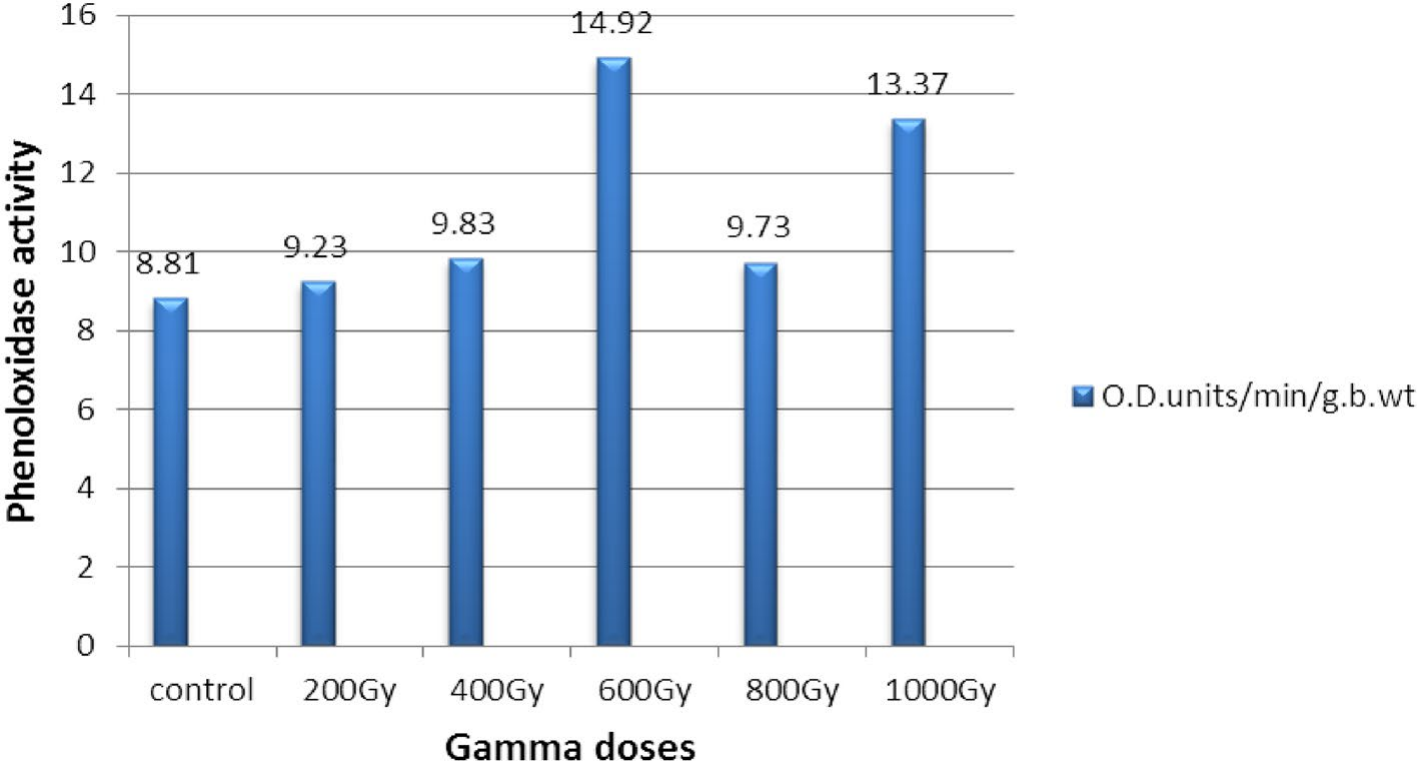
Effect of gamma radiation on phenoloxidase activity in larvae of the *Ephestia cautella.*

The experiment's findings on the effects of radiation on phenoloxidase in larvae of the *E. cautella* indicate variability of the response of enzyme activity to irradiation (Table 2 and Fig. 2). Phenoloxidase activity in irradiated larvae with doses of 200, 400 and 800 Gy was similar to that of the control, while at doses of 600 and 1000 Gy the Phenoloxidase activity increased to 14.92 and 13.37 O.D.units respectively compared to 8.81 O.D.units in the control.

**Table 2:** Phenoloxidase activity in unirradiated and irradiated larvae of *Ephestia cautella*.

Dose (Gy)	(O.D. units/min/g.b.wt)
Control	8.81 ± 0.31^a^
200	9.23 ± 0.15^ab^
400	9.83 ± 0.38^b^
600	14.92 ± 1.05^d^
800	9.73 ± 0.5^ab^
1000	13.37 ± 0.51^c^
F value	69.15
L.S.D. 5%	0.95

*Common letter following the mean indicates no significant difference between means in a column.

### Effect of gamma radiation on the cuticle of *Ephestia cautella* larvae:

**Figure 3:**
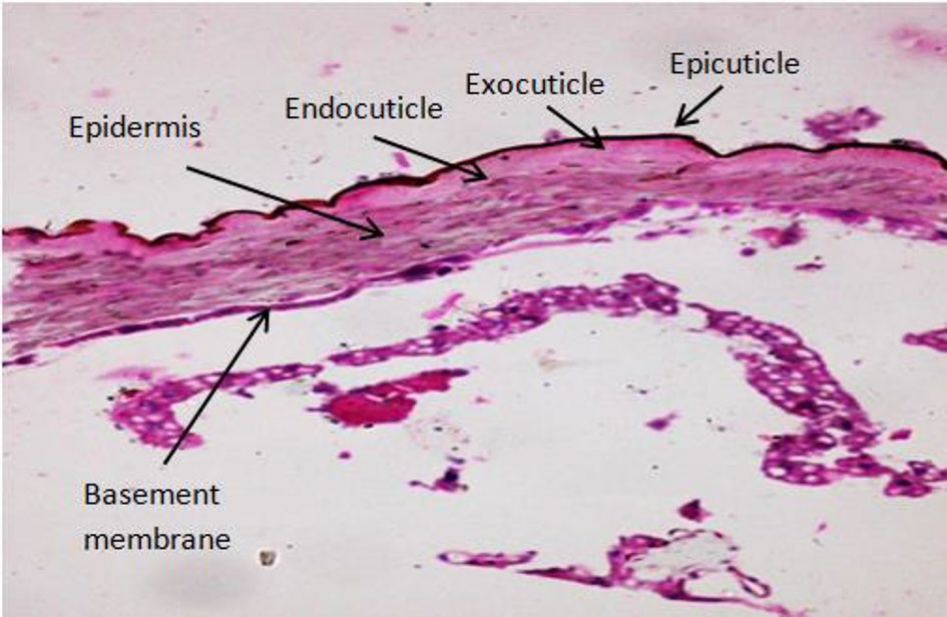
Cross section of *Ephestia cautella* 4th instar larva showing the different cuticle layers, epidermis and basement membrane. (H&E X 400).

In order to determine which cuticle layers contain melanin granules and how the various cuticle layers are arranged and shaped, histological examinations on the abdominal cuticles of *E. cautella* larvae in their fourth instar were conducted. Transverse sections of the abdominal cuticle samples demonstrated that body wall consists of 3 layers (cuticle, epidermis and basement membrane). Cuticle is the thickest, outermost integument layer secreted by the epidermis. It is composed of two layers (epicuticle and procuticle) which the procuticle consists of exocuticle and endocuticle layers (Fig. 3).

**Figure 4:**
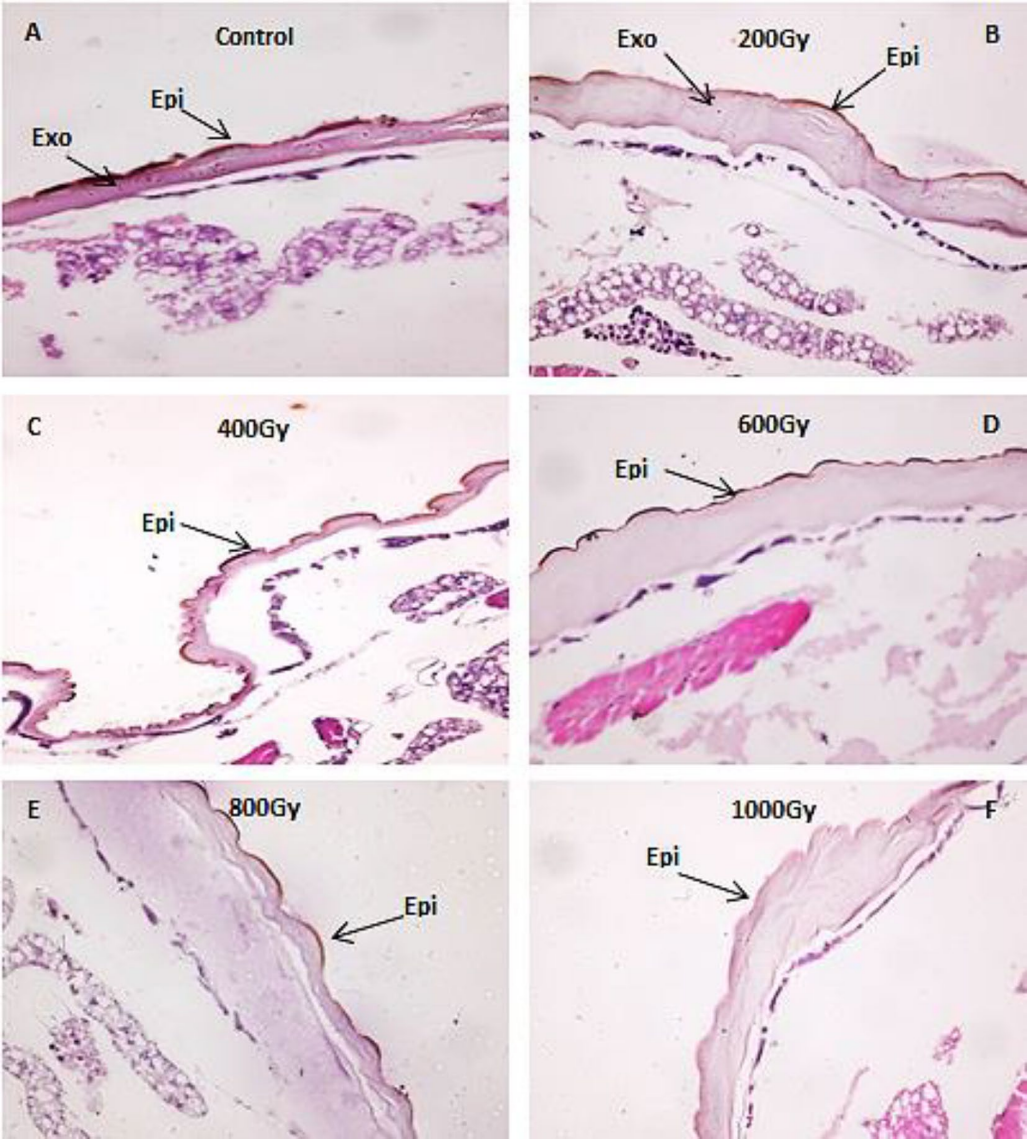
Cross section of *Ephestia cutella* 4th instar larvae showing the effect of gamma radiation on the cuticle and the arrows indicate the layers that contain melanin pigment granules. (Epi: Epicuticle; Exo: Exocuticle). (H&E X 400).

The microscopic examination of the unirradiated larvae of *E. cautella* made it evident that brown melanin pigment granules had been deposited in the epicuticle and exocuticle layers of the cuticle (Fig. 4A). When larvae were exposed to gamma radiation doses, the amount of melanin pigment gradually decreased until it eventually vanished from the cuticle layers at higher doses (1000 Gy).

At dose 200 Gy the larvae showing faint brown homogeneously melanin pigment layer limited to epicuticle layer of cuticle. The exocuticle became a thin layer of brown homogeneously melanin pigment spread out in this layer (Fig. 4B). When the dose was increased to 400 and 600 Gy, the melanin granules only appeared in the epicuticle layer and there was a extensive concentration of brown granules deposited, especially at the tips (Fig. 4C and D). Larvae exposed to 800 Gy revealed a weak dark melanin layer restricted to certain regions of the epicuticle layer, whereas at the higher dose of 1000 Gy, the melanin granules vanished from all layers (Fig. 4E and F).

An ideal method for detecting irradiated insects should be: (1) specific for irradiation and not influenced by other processes, (2) accurate and reproducible, (3) have a detection limit below the minimum dose likely to be applied to an agricultural commodity as a quarantine treatment, (4) applicable to a range of pests, (5) quick and easy to perform, and (6) capable of providing an estimate of irradiation dose^29^. The melanin test used here to detect irradiated larvae of stored pests do not fulfill these requirements. The tests often produced inconsistent results because (a) irradiation does not completely prevent melanization in pest larvae, and (b) untreated larvae, cold-killed and examined at room temperature, often show incomplete melanization we observed untreated insects that showed a clear lack of melanization (natural color) or were melanized only partially, often as those that have been irradiated.

According to Nation et al.^10^, ionizing radiation may strongly inhibit melanization in some pest larvae when doses are applied at a sufficient level because it prevents the production of one or more melanization-related enzymes rather than altering the enzymatic reactions that lead to melanization. They noted that after freezing and thawing, control larvae of the Caribbean fruit fly *Anastrepha suspensa* quickly turned brown. First instars of the pest irradiated with > 20 Gy and examined for whole melanization as late third instars faded to show typical melanization and their results were supported by Mansour and Franz^30^ However, their papers do not provide data on the percentage of irradiated and unirradiated larvae that went through the process, and on the percentage of their bodies encompassed by melanization. Results of the present paper indicate clearly that gamma radiation of larvae of stored product pests inhibit their melanization, but do not completely prevent this process.

Banasik-Solgala and Stanislaw^31^ found that treated and untreated insects had considerably differed significantly between levels of melanization. It decreased with increasing dose after treatment in old larvae of the Indian meal moth, *Plodia interpunctella*, the Mediterranean flour moth, *Ephestia (Anagasta) kuehniella*, and the almond moth, *Cadra cautella*. Ignatowicz and Ibrahaim^32^ reported that when irradiation young larvae of the confused flour beetle, *Tribolium confusum*, the melanization was reduced in first week. Great variation of melanin pigmentation in the untreated old larvae partially obscured the effects of gamma radiation on this process. However, the melanization was considerably reduced in all the experiments involving old larvae. Ignatowicz and Katarzyna^33^ the first and second instar larvae of the Khapra beetle, *Trogoderma granarium* experienced a considerable inhibition of the melanization process following irradiation treatment with doses ranging from 100 to 500 Gy, which killed by freezing on the 1^st^ and 2^nd^ week after irradiation. Also irradiation inhibited significantly the melanization of the 4^th^ instar larvae after their death; however, due to the wide variation in how the melanization process reacts to irradiation treatment, the change in melanization of Khapra beetle larvae cannot be utilized to indicate prior exposure of these insects to irradiation. Since phenoloxidase activity rises with larval development and reaches a maximum just before pupation, larvae appear to have a high quantity of the enzyme^30,34^. Being saturated with the enzyme, the mature larvae do not react so clearly to irradiation as those larvae that was irradiated as immature larvae^4,10,30^. This may explain the variable results obtained with old larvae.

The Almond moth, *E. cautella*, showed extremely varied phenoloxidase enzyme activity for both control and irradiated larvae, suggesting that other, as-yet unidentified, factors may affect the enzyme's activity. These factors must be identified. As a result, a test based on phenoloxidase activity cannot yet be used with sufficient accuracy in the quarantine process to identify unirradiated insects that are pests of stored goods.

The light microscope photos demonstrated how the larvae's cuticle changed color during the melanization process. Our findings suggested that the deposition of black melanin-like granules found in the epicuticle and exocuticle layers are responsible for the black cuticle color of *E. cautella* larvae. This has been demonstrated in the lepidopteran larval cuticles^35^. However, a prior investigation revealed that the black mutant larva of the *Bicyclus anynana* lacked melanin granules in the epicuticle, and that the epicuticle appeared to have a distal homogenous coating of dark pigment^36^. Accordingly, the methods of black pigmentation in the larval cuticle of various Lepidopteran species may differ dependent on these structural characteristics^35,36^. Melanin deposits in the cuticle are primarily responsible for the black color of the body. Melanin is produced by epidermal cells through the melanin synthesis pathway^37,38^.

## Conclusion

The recommended radiation dose for quarantine therapy just delays reaching the adult stage, but only prevents completion of development to the adult stage. Consequently, if a living larva is detected in shipment, it is very important to determine if it has been irradiated. According to the results of the current study, it is possible to quickly and easily determine if larvae had previously been exposed to ionizing radiation by looking at how gamma radiation affects the melanization process in larvae killed by freezing.

## Data Availability

All data generated or analyzed during this study are included in this published article.
